# The alterations in peripheral lymphocyte subsets predict the efficacy and prognosis of immune checkpoint inhibitors in hepatocellular carcinoma

**DOI:** 10.7150/jca.88101

**Published:** 2023-09-11

**Authors:** Qu Xie, Can Hu, Cong Luo

**Affiliations:** 1 Department of Hepato-Pancreato-Biliary & Gastric Medical Oncology, Zhejiang Cancer Hospital, Hangzhou Institute of Medicine (HIM), Chinese Academy of Sciences, Hangzhou, Zhejiang, 310022, China.; 2 Postgraduate training base Alliance of Wenzhou Medical University (Zhejiang Cancer Hospital), Hangzhou, Zhejiang, 310022, China.; 3 The Second School of Clinical Medicine of Zhejiang Chinese Medical University, Hangzhou, 310053, China.; 4 Zhejiang Cancer Hospital, Hangzhou Institute of Medicine (HIM), Chinese Academy of Sciences, Hangzhou, Zhejiang, 310022, China.

**Keywords:** immune checkpoint inhibitor (ICI), hepatocellular carcinoma (HCC), biomarker, peripheral blood lymphocyte subsets

## Abstract

**Background:** Immune checkpoint inhibitor (ICI) treatments are promising therapies for hepatocellular carcinoma (HCC) patients. However, not all HCC patients benefit from immunotherapy. Therefore, it is urgent to explore markers for the clinical efficacy and prognosis of immunotherapy for liver cancer. This study aimed to investigate changes in peripheral blood lymphocyte subsets after immunotherapy and to assess their predictive and prognostic value.

**Methods:** Sixty-one patients with advanced HCC were enrolled. Peripheral blood samples were collected from HCC patients before and after ICI treatment, and lymphocytes were detected by flow cytometry. The rank sum test, chi-square test, Kaplan‒Meier curve, and Cox regression model were used to determine the relationship between the changes in the percentages of peripheral blood lymphocyte subsets and clinicopathological characteristics, clinical efficacy, progression-free survival (PFS) and overall survival (OS).

**Results:** After ICI treatment, the percentage of CD3^+^CD8^+^ T cells increased, and the percentage of B cells decreased. The changes in memory T cells percentages varied according to different immune efficacy groups. Age, history of hepatitis B infection, first-line therapy, and distant metastasis influenced the proportion of peripheral blood lymphocyte subsets in patients with advanced HCC. Furthermore, univariate analysis demonstrated that high percentage changes in the natural killer (NK) cells percentage change predicted longer PFS and OS.

**Conclusions:** ICI treatment alters the percentage of peripheral blood lymphocyte subsets in immunotherapy-treated HCC patients. Changes in the proportion of lymphocyte subsets are influenced by variances in immunological response and clinicopathological features. A high degree of NK cells percentage change in HCC patients treated with ICI represents an independent prognostic predictor.

## Background

Hepatocellular carcinoma (HCC) is the fifth most common cancer and the third leading cause of cancer death worldwide. Chinese patients with liver cancer account for more than 40% of the world's incidence^1^, ^2^. Because the early symptoms are not obvious, the degree of malignancy is high, and the rate of development and deterioration is fast, 75% of HCC patients have developed advanced manifestations such as distant metastasis at the time of diagnosis and cannot benefit from radical surgical resection^3^. Many advances have been made in the clinical diagnosis and treatment of HCC in recent years, but the 5-year overall survival (OS) rate of HCC patients is only 12%^4^. How to apply existing treatment methods to improve the cure rate and prolong the survival time of liver cancer in a planned and reasonable manner has become an urgent problem to be solved in the field of advanced liver cancer treatment.

In recent years, immune checkpoint inhibitor (ICI) treatment has promoted considerable survival benefits to patients with advanced HCC, making long-term survival possible^5^. Immunotherapy with ICI blocks the specific binding of PD-1 on the surface of immune cells, such as T cells and B cells, to PD-L1 on the surface of tumour cells and blocks the PD-1/PD-L1 pathway through programmed death-1 (PD-1) antibodies or ligand (PD-L1) antibodies, thereby relieving immunosuppression and exerting tumour-killing effects^6^. The compelling exploration of immunotherapy has broken through the bottleneck of liver cancer treatment for over a decade. The IMbrave 150 study showed that compared with sorafenib, the combination of atezolizumab and bevacizumab significantly prolonged OS (19.2 months vs. 13.4 months, hazard ratios (HR) =0.66,* P* = 0.0009) and progression-free survival (PFS) (6.9 months vs. 4.3 months, HR=0.65, *P* = 0.0001) in patients with advanced unresectable liver cancer^7^. Although immunotherapy has made outstanding progress in the treatment of HCC, it has a low response rate (12-20% effective rate with single-agent immunization^8^-^10^ and 27-46% effective rate with combination therapy^11^-^14^). Moreover, these treatments are expensive, which significantly limits the application of ICI. Therefore, finding appropriate immune efficacy markers, screening the population that would benefit from immunotherapy, and effectively improving the effective rate of immune combination therapy are urgent clinical problems to be solved.

Because lymphocytes in peripheral blood have the significant advantages of low invasiveness and real-time efficacy monitoring, an increasing number of studies have investigated their predictive and prognostic value in cancer patients in recent years^15^, ^16^. For example, in 16 HCC patients treated with nivolumab, patients with progressive disease were more likely to have monocytes with increased PD-L1 positivity at 4 weeks (*P* = 0.020) or 6 weeks (*P* = 0.008) of treatment^17^. In addition, patients with increased active circulating CD8^+^ T cells and downregulation of neutrophil-related markers during pembrolizumab treatment had better clinical benefits^18^. However, the value of peripheral blood immune cells and their subsets in predicting the efficacy and prognosis of immunotherapy in patients with advanced HCC needs further exploration.

Therefore, in this study, we assessed changes in peripheral blood lymphocyte subsets before and after immunotherapy and their relationship with clinical efficacy and prognosis in patients with advanced HCC treated with ICI.

## Materials and methods

### Patients

This study included Barcelona Clinic Liver Cancer (BCLC) B or C patients who received ICI treatment at Zhejiang Cancer Hospital from January 2019 to December 2021. The inclusion criteria were as follows: 1 age > 18 years; 2 pathologically or clinically diagnosed HCC patients with BCLC stage B or C disease; 3 Eastern Cooperative Oncology Group performance status (ECOG) score of 0 to 2 and Child‒Pugh grade A or B; 4 complete lymphocyte subsets and clinicopathological information; and 5 at least one measurable target lesion on baseline computed tomography (CT) or magnetic resonance imaging (MRI). Patients with a history of autoimmune diseases or with tumours other than HCC were excluded.

The Zhejiang Cancer Hospital's Ethics Committee granted approval for this retrospective study (IRB-2023-87), waiving the requirement for informed patient consent in adherence with the guidelines of the Declaration of Helsinki.

### Treatment

The study cohort comprised individuals undergoing ICI treatment as a sole therapy or in conjunction with targeted therapies. Patient groups were administered ICI regimens, including atezolizumab (10 patients), camrelizumab (17 patients), tislelizumab (12 patients), and cedilimumab (22 patients). Intravenous administration was employed for atezolizumab at a dose of 1200 mg every three weeks, while the remaining ICI drugs were administered intravenously at 200 mg every three weeks. Among the 50 patients undergoing combined targeted therapies, 6 received a combination of the anti-vascular endothelial growth factor drug bevacizumab. In comparison, 42 patients received a combination of anti-tyrosine kinase inhibitors (including 8 cases of apatinib, 21 cases of lenvatinib, 4 cases of regorafenib, and 11 cases of sorafenib). Established clinical practice criteria guided the selection of targeted therapeutic regimens.

### Data collection and response assessment

The collected clinicopathological data were analysed, including sex, age, hepatitis B infection history, Child‒Pugh class, pretreatment alpha-fetoprotein (AFP) level, BCLC stage, ECOG score, line of therapy, combination with targeted therapy, combination with local therapy, and distant metastasis.

Peripheral venous blood was collected one week before ICI treatment and at the optimal response time. Various lymphocyte subsets were assessed, including CD3^+^ T cells, CD3^+^CD4^+^ T cells, CD3^+^CD8^+^ T cells, CD4/CD8 ratio, natural killer (NK) cells (CD3^-^CD56^+^), B cells (CD3^-^CD19^+^), natural killer T (NKT) cells (CD3^+^CD56^+^), Ts cells (CD4^+^CD45RA^+^), memory T cells (CD4^+^CD45RO^+^), activated T cells (CD45RA^+^CD45RO^+^) and activated CD8^+^ cells (CD8^+^CD38^+^).

Enhanced CT or enhanced MRI for clinical efficacy assessments was performed 6-8 weeks after immunotherapy and every 2-3 months thereafter. The Response Evaluation Criteria in Solid Tumours version 1.1 (RECIST 1.1) was used to assess the clinical response. The best overall response (BOR) during the whole ICI treatment phase was classified as complete response (CR), partial response (PR), stable disease (SD), or progressive disease (PD). Patients with response assessments of CR, PR, and SD were assigned to the responder group, and those with PD were assigned to the nonresponder group. PFS refers to the time interval from the start of treatment to tumour progression or death from any cause. In contrast, OS refers to the time interval from the beginning of treatment to death from any reason or the last follow-up.

### Flow cytometry

The flow experiment steps were as follows: 1 three flow cytometry tubes were required for each peripheral blood sample and labelled Tube 1, Tube 2, and Tube 3. 2 Different premixed monoclonal antibodies were added to the three tubes, and 50 μL of anticoagulated blood was added to each tube. 3 The samples were mixed well and incubated at room temperature in the dark for 15 min. 4 Then, 500 μL of haemolysin was added and mixed well with vortex shaking, and the samples were placed in the dark for 15 min. 5 One millilitre of sheath fluid was added to terminate haemolysis. 6 The blood samples were centrifuged at 1500 rpm for 5 min, and the supernatant was discarded. 7 Then, 500 μL of sheath fluid was added to resuspend the sample, and the percentages of peripheral blood lymphocyte subsets were measured using a Beckman Coulter Cytometer FC500 flow cytometer. Detailed operating procedures and associated materials are provided in Additional file 1.

### Statistical analysis

Continuous variables are presented as medians, and categorical data are presented as counts and percentages. Comparisons between groups were made using the Wilcoxon two-independent-samples rank-sum test, and comparisons within groups were made using the Wilcoxon paired-samples rank-sum test. Categorical variables were compared using the chi-square test. In predictive analysis, for each lymphocyte subset, the median was set as a cut-off, and each subset was divided into two subgroups (high and low). Univariate Cox proportional hazards regression models were used to assess the effect of clinical history information and lymphocyte subsets (expressed as HR and their corresponding 95% confidence intervals (CI)) on PFS and OS. PFS and OS were estimated with the Kaplan‒Meier method and analysed using the log-rank test. All statistical tests were two-sided, and P values <0.05 were considered statistically significant. All analyses were conducted using SPSS 25.0 software (International Business Machines Corporation, Armonk, NY, USA) and GraphPad Prism 9.0 (GraphPad Software Corporation, San Diego, CA, USA).

## Results

### Participant characteristics

**Figure 1:**
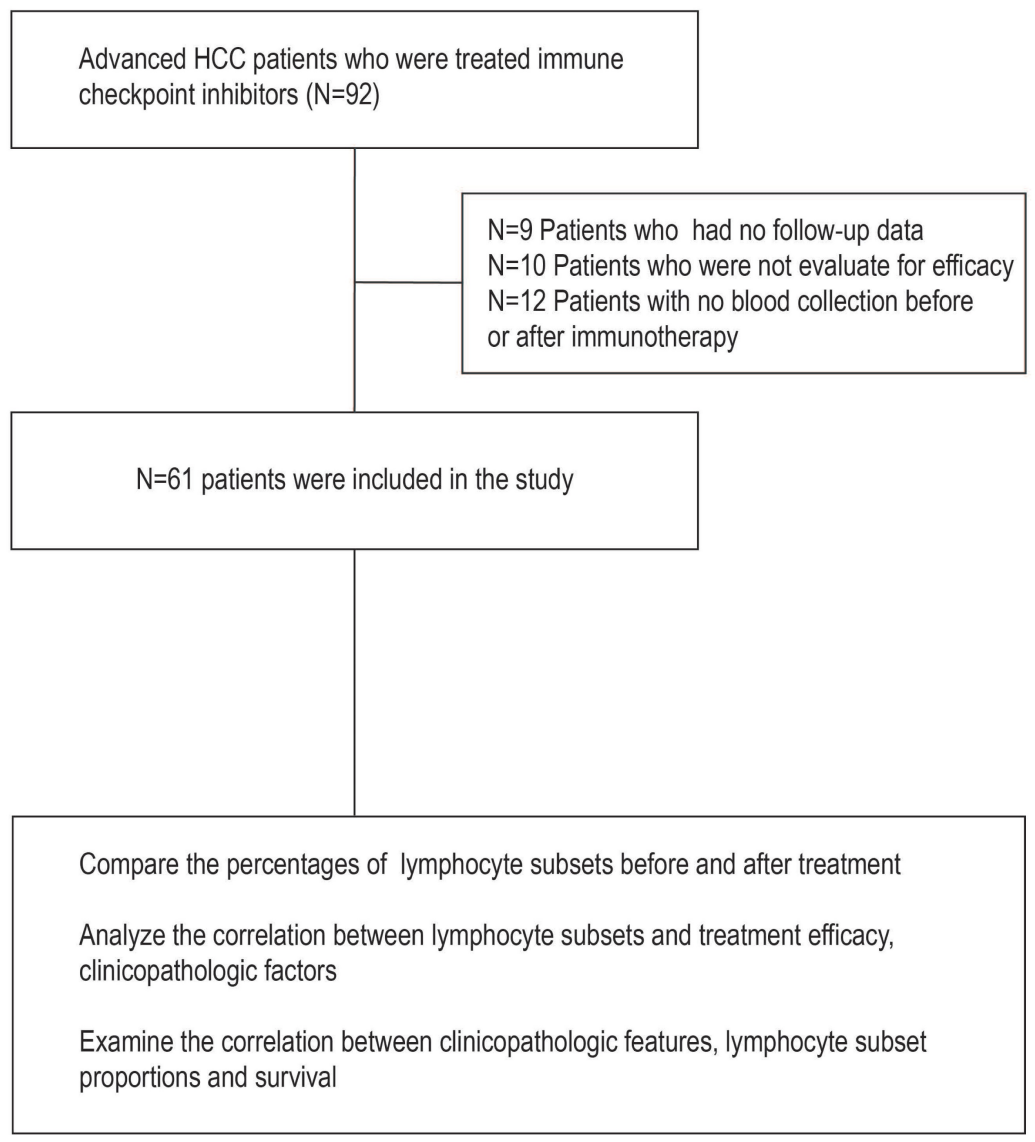
** Workflow of this study. Abbreviation:** HCC, hepatocellular carcinoma.

From 01 January 2019 to 31 December 2021, 61 patients with advanced HCC met the inclusion and exclusion criteria (Figure 1). The baseline characteristics of all patients are shown in Table 1. Forty-two (68.9%) patients were 60 years or younger, and 51 (83.6%) were male. Of these patients, the majority had hepatitis B infection (46, 75.4%). Fifty-three (86.9%) patients had Child‒Pugh class ratings of A, while 8 (13.1%) patients had class ratings of B. In addition, 26 (42.6%) patients had an AFP level of ≥ 400 IU/ml at baseline. The BCLC stage was C in 50 (82.0%) patients and B in 11 (18.0%). In addition, 43 patients (70.5%) had an ECOG score of 1, and 18 others (29.5%) had an ECOG score of 0. Forty-four patients (72.1%) received first-line therapy, 50 patients (82.0%) were treated with concomitant targeted treatment, and 24 patients (39.3%) were treated with concomitant local treatment. In addition, 27 patients (44.3%) had distant metastasis.

### Efficacy

As of 14 November 2022, the median follow-up time was 20.1 months (95% CI: 16.0 to 24.2 months). The median PFS and OS were 8.3 months (95% CI: 5.5 to 11.1 months) and 19.4 months (95% CI: 6.0 to 32.8), respectively. The objective response rate (ORR) was 32.79% [20/61, 0 CR, 20 PR]. The disease control rate (DCR) was 78.69% [48/61, 0 CR, 20 PR, 28 SD].

### Changes in lymphocyte subsets in peripheral blood after ICI treatment

**Figure 2:**
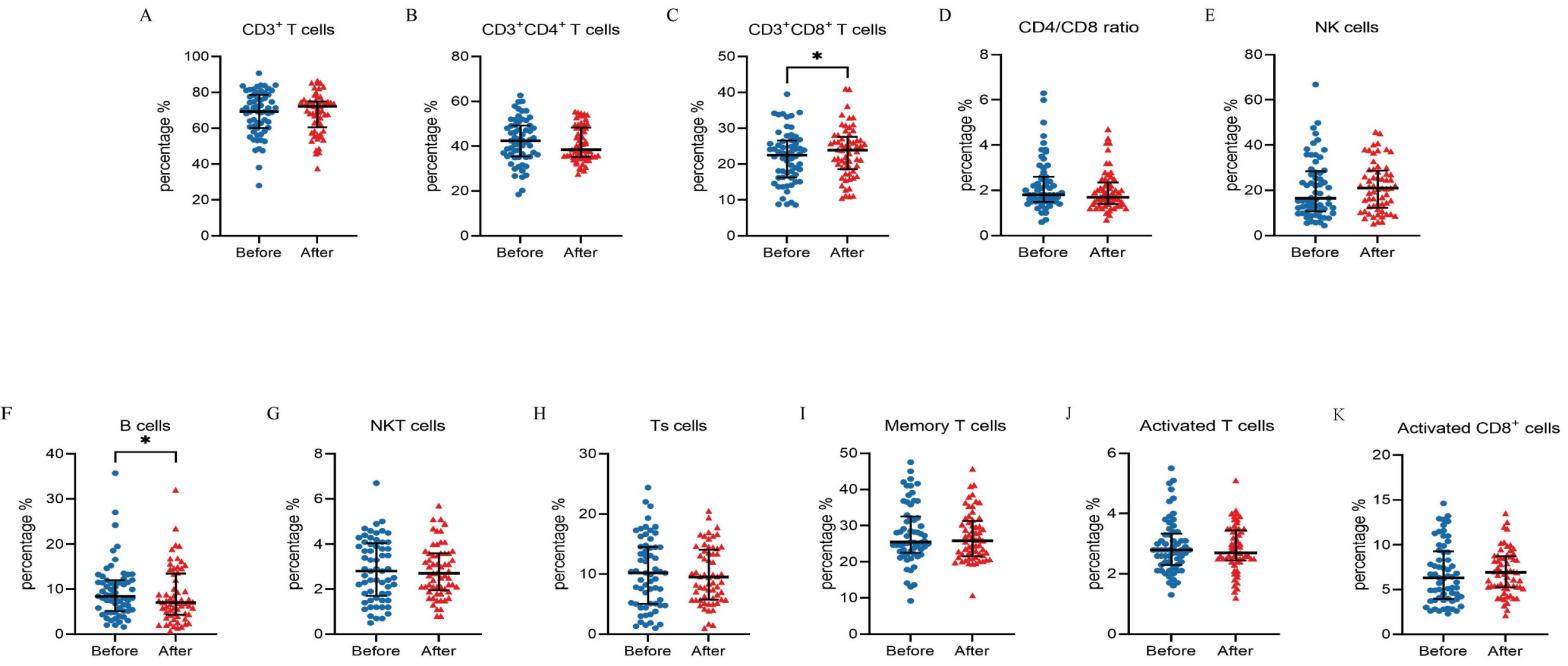
** Percentage of different subpopulations of peripheral blood lymphocytes before and after Immune checkpoint inhibitor (ICI) treatment.** (A) CD3^+^ T cells, (B) CD3^+^CD4^+^ T cells, (C) CD3^+^CD8^+^ T cells, (D) CD4/CD8 ratio, (E) natural killer (NK) cells (CD3^-^CD56^+^), (F) B cells (CD3^-^CD19^+^), (G) natural killer T (NKT) cells (CD3^+^CD56^+^), (H) Ts cells (CD4^+^CD45RA^+^), (I) memory T cells (CD4^+^CD45RO^+^), (J) activated T cells (CD45RA^+^CD45RO^+^), and (K) activated CD8^+^ cells (CD8^+^CD38^+^). *, P<0.05.

The proportions of changes in peripheral blood lymphocyte subsets after ICI treatment are shown in Figure 2 and Table 2. The results showed an increase in the percentage of CD3^+^CD8^+^ T cells (*P* = 0.034) and a decrease in the percentage of B cells (*P* = 0.036) after immunotherapy compared with baseline (Figure 2 and Table 2). This suggests an effect of ICI treatment on peripheral lymphocyte subsets.

### Correlation between the proportion change in lymphocyte subsets in peripheral blood and efficacy after ICI treatment

After immunotherapy, 48 patients were classified as responders (0 CR, 20 PR, 28 SD), and 13 were classified as nonresponders. In addition, the changes in the percentage of lymphocytes were divided into decreased groups and increased groups. The results showed that the effective rate was 69.23% in the decreased memory T cells group and 77.42% in the increased memory T cells group (Table 3). There was a significant difference in the effective rate between the two groups (*P* = 0.016).

### Factors influencing lymphocyte subsets

We collected clinicopathological information before ICI treatment and analysed its effect on the percentage of changes in different lymphocyte subsets (Table 4). The results showed that the percentage of activated T cells and activated CD8^+^ cells decreased in HCC patients over 60 years of age after ICI treatment compared with patients under 60 years (Table 4). In addition, hepatitis B infection history may influence the changes in CD3^+^ T cells and activated CD8^+^ T cells after ICI treatment (Table 4). The percentage of CD3^+^ T cells decreased and that of activated CD8^+^ T cells increased in patients with a history of hepatitis B infection after ICI treatment compared with patients without a history of hepatitis B infection (Table 4). Moreover, compared with HCC patients who did not receive first-line therapy, those who did receive first-line therapy after ICI therapy had decreased proportions of B cells and memory T cells (Table 4). Changes in the percentages of CD3^+^CD4^+^ T cells, the CD4/CD8 ratio, and B cells were associated with distant metastasis. Compared with HCC patients without distant metastasis, HCC patients with distant metastasis after ICI treatment had decreased percentages of CD3^+^CD4^+^ T cells and CD4/CD8 ratio, while the percentage of B cells increased (Table 4). These results suggest that age, history of hepatitis B infection, first-line therapy, and distant metastasis may influence the change in the percentage of peripheral blood lymphocyte subsets after ICI treatment.

### Correlation between clinicopathological features, baseline lymphocyte subset percentages or changes in peripheral blood lymphocyte subset percentages and survival

**Figure 3:**
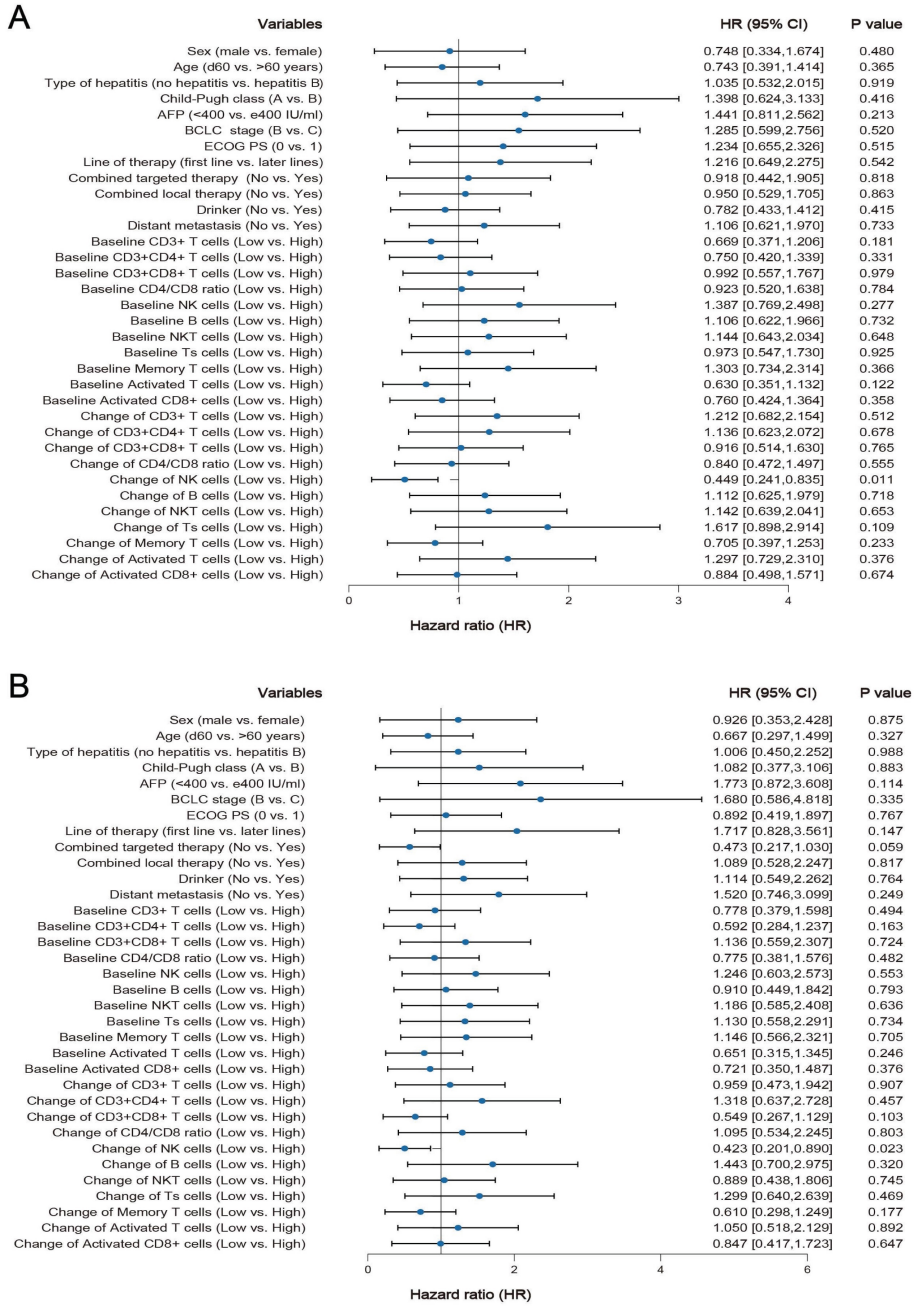
** Univariate Cox regression analysis of progression-free survival (PFS) (Figure 3A) and overall survival (OS) (Figure 3B) for clinical characteristics and peripheral blood lymphocyte subsets.** A median cell percentage was employed as the threshold to distinguish between low and high levels.

**Figure 4:**
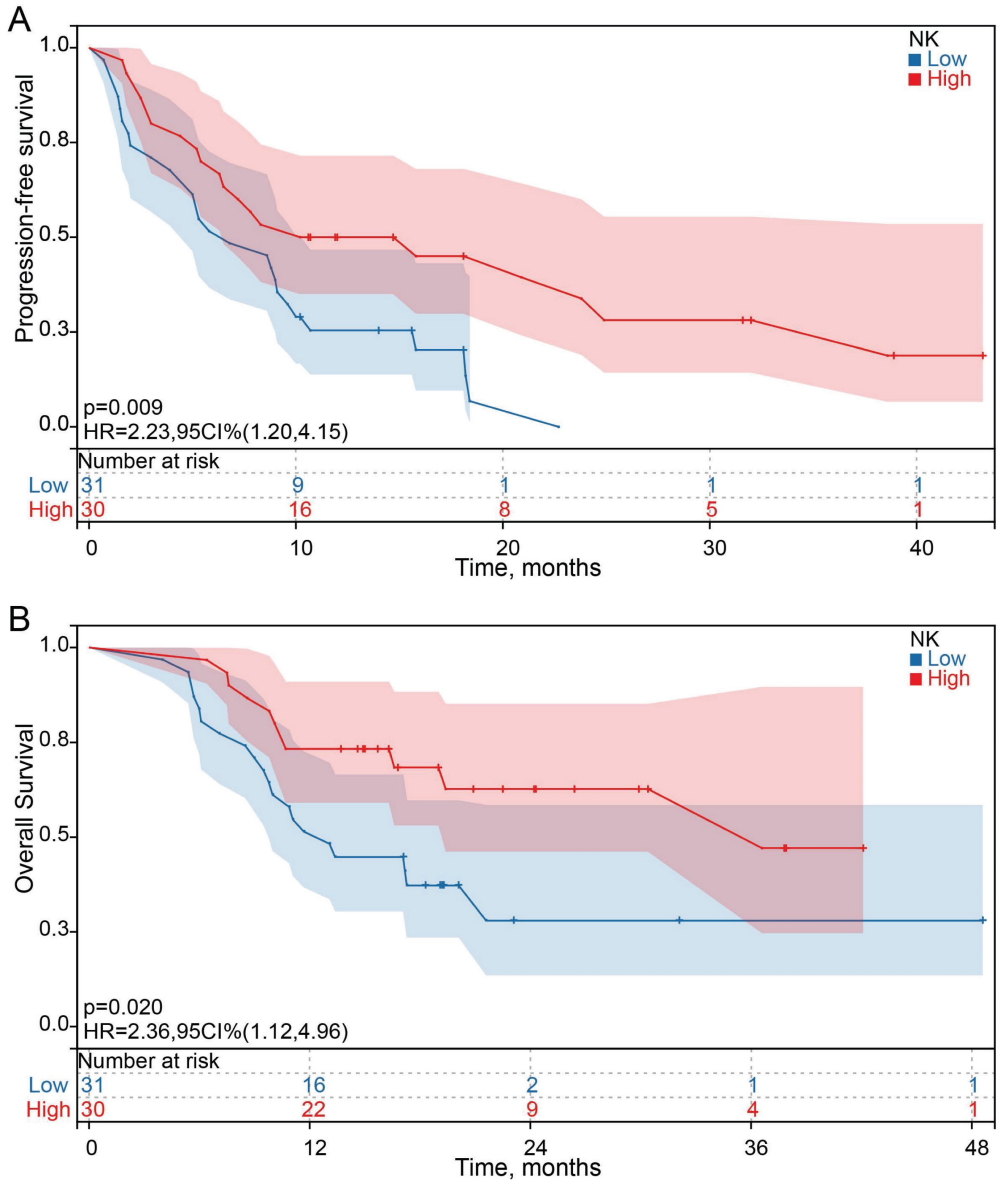
Kaplan-Meier survival curves for the association between the changes in NK cells proportions and PFS and OS.

Among the 61 HCC patients, the median PFS was 8.3 months (95% CI: 5.5 to 11.1 months), and the median OS was 19.4 months (95% CI: 6.0 to 32.8). Univariate analysis showed that all clinicopathological characteristics and the percentage of peripheral blood lymphocyte subsets before ICI treatment were not prognostic factors for PFS and OS in HCC patients treated with ICI. However, the changes in the percentage of NK cells after ICI treatment were independent prognostic factors for PFS (*P* = 0.011) and OS (*P* =0.023) in HCC patients treated with ICI, and the changes in the percentage of other lymphocyte subsets were not prognostic factors for PFS and OS (Figure 3 A and B). Survival analysis was performed to further determine the predictive value of ICI treatment based on peripheral blood lymphocyte subsets in HCC patients. The results showed that HCC patients with a high percentage of NK cells had longer OS and PFS than HCC patients with a low percentage of NK cells after ICI treatment, which was consistent with the univariate Cox regression analysis results (Figure 4).

## Discussion

In this study, 61 HCC patients treated with ICI were recruited. The results showed an increased CD3^+^CD8^+^ T cells percentage and a decreased B cells percentage after immunotherapy. Moreover, there were statistically significant differences in the changes in percentages of memory T cells in different immune efficacy groups. In addition, changes in the percentages of activated T cells and activated CD8^+^ T cells after ICI treatment were age-related; changes in the proportions of CD3^+^ T cells and activated CD8^+^ cells were related to hepatitis B infection history; changes in the proportions of B cells and memory T cells were associated with first-line treatment; and changes in the percentages of CD3^+^CD4^+^ T cells, the CD4/CD8 ratio and B cells were associated with distant metastasis. Additionally, univariate analysis and survival analysis showed that high percentage changes of NK cells predicted good PFS and OS in ICI-treated HCC patients.

In the development of tumours, tumour cells bind to PD-1 on the surface of T cells by overexpressing PD-L1, inactivating T cells and making them unable to kill tumour cells promptly, resulting in immune escape of tumour cells^6^. This study showed that the CD8^+^ T cells percentage increased after ICI treatment. This observation of systemic proliferation of CD8^+^ T cells after ICI treatment is consistent with the results reported by Fei et al. and Alexander et al.^19^, ^20^. It may be possible that blocking the PD-1/L1 pathway with ICI treatment relieves immunosuppression, restores the viability of CD8^+^ T cells, and induces their activation and proliferation^21^. Moreover, blockade of the PD-1/PD-L1 pathway increases B cells activation, proliferation, and immunoglobulin secretion^22^. Nevertheless, we found that the percentage of B cells decreased after immunotherapy. It may be that B cells undergo isotype switching upon activation by antigens^19^, ^23^.

According to the expression differences of cell surface molecules, CD4^+^ T cells were divided into Ts cells (CD4^+^ CD45RA^+^) and memory T cells (CD4^+^ CD45RO^+^)^24^, ^25^. Compared with Ts cells, memory T cells reencounter the same antigen and respond and proliferate faster^26^. Furthermore, we analysed the correlation between changes in lymphocyte subset percentages after immunotherapy and clinical efficacy. The results showed statistically significant differences in the changes in the percentages of memory T cells in different immune efficacy groups. This finding suggests that differences in memory T cells responses may contribute to differential immune efficacy. Interestingly, in Peng Yang et al.'s study of the predictive role of the peripheral CD4^+^ memory ratio in non-small cell carcinoma, patients with increased memory CD4^+^ T cells may have a poor PFS (*P* = 0.138)^27^. Memory CD4^+^ T cells may proliferate less than primary reactive cells when they reencounter antigens in peripheral blood and do not elicit a robust immune response^28^, ^29^. This hypothesis requires further exploratory validation.

With age, the body's immunity and immune cells continue to decline, and the ability of the immune system to activate against tumours also decreases^30^. We found that the percentages of activated T cells and activated CD8^+^ cells were inversely correlated with age, which is consistent with previous studies^29^, ^31^. The immunopathological mechanism of hepatitis B infection is mainly T cells immunity, including CD3^+^ T cells, CD4^+^ T cells, CD8^+^ T cells, and activated cells, in which the immune response to viral antigens is associated with viral elimination and pathogenesis^32^, ^33^. This study showed that patients with a history of hepatitis B infection had decreased percentages of CD3^+^ T cells and increased percentages of activated CD8^+^ T cells. These results suggest that hepatitis B infection causes disturbances in T cells subsets.

Increasing evidence suggests that peripheral blood lymphocyte subsets have predictive and prognostic value in cancer patients^34^-^36^. However, predictive biomarkers for immunotherapy in HCC patients need further exploration. In this study, we showed that a high NK cells percentage predicted a better prognosis in HCC patients. However, none of the other peripheral blood lymphocyte subsets were prognostic factors for PFS and OS in HCC patients with ICI treatment. Therefore, prospective studies are needed to recruit more patients to validate the further association between peripheral blood lymphocyte subsets and PFS and OS.

This study had several limitations. First, its retrospective nature and limited number of patients may have introduced case selection bias and narrow results, and prospective studies are needed to recruit more patients to avoid this bias. Second, whether combined with local therapy, different ICI drugs may be potential confounders. Finally, the follow-up period of this study was a relatively short median of 20.1 months, and future studies will need to extend the observation time and treatment cycles, longitudinally monitor changes in lymphocyte subsets, and further explore their correlation with PFS and OS.

## Conclusions

ICI treatment induced changes in the percentages of peripheral blood lymphocyte subpopulations. The changes in the proportion of memory T cells were significantly different among different immune efficacy groups. Significant percentage changes in NK cells were associated with longer PFS and OS. Therefore, peripheral blood lymphocyte subsets may serve as valuable markers for the efficacy and prognosis of immunotherapy in advanced HCC patients.

## Supplementary Material

Supplementary information.

## Figures and Tables

**Table 1:** Clinical and pathological characteristics.

	N	%
**Sex**		
Male	51	83.6
Female	10	16.4
**Age**		
≤60	42	68.9
>60	19	31.1
**Type of hepatitis**		
No hepatitis	15	24.6
Hepatitis B	46	75.4
**Child-Pugh class**		
A	53	86.9
B	8	13.1
**AFP**		
<400(IU/ml)	35	57.4
≥400(IU/ml)	26	42.6
**BCLC stage**		
B	11	18.0
C	50	82.0
**ECOG PS**		
0	18	29.5
1	43	70.5
**Line of therapy**		
First line	44	72.1
Later lines	17	27.9
**Combined targeted therapy**		
No	11	18.0
Yes	50	82.0
**Combined local therapy**		
No	37	60.7
Yes	24	39.3
**Drinker**		
No	35	57.4
Yes	26	42.6
**Distant metastasis**		
No	34	55.7
Yes	27	44.3

**Abbreviations:** AFP, alpha-fetoprotein; BCLC, Barcelona Clinic Liver Cancer; ECOG PS, Eastern Cooperative Oncology Group Performance Status.

**Table 2:** Percentage of different subpopulations of peripheral blood lymphocytes before and after ICI treatment.

Lymphocyte subset	Percentage of HCC patients		
	Before (median (IQR))	After (median (IQR))	P
CD3^+^ T cells	69.3 (60.2,78.7)	72.2 (60.5,75.1)	0.874
CD3^+^CD4^+^ T cells	42.4 (35.4, 49.3)	38.4 (35.2,48.3)	0.653
CD3^+^CD8^+^ T cells	22.5 (16.3 26.6)	23.9 (18.6,27.6)	0.034
CD4/CD8 ratio	1.8 (1.5, 2.6)	1.7 (1.4,2.4)	0.098
NK cells	16.5 (10.7, 28.4)	21.0 (12.3,28.8)	0.367
B cells	8.4 (5.1,12.0)	7.0 (4.3,13.5)	0.036
NKT cells	2.8 (1.7, 4.1)	2.7 (2.0,3.6)	0.707
Ts cells	10.2 (5.1, 14.6)	9.5 (5.8,14.1)	0.236
Memory T cells	25.3 (23.3, 33.0)	24.7 (22.5,31.6)	0.411
Activated T cells	2.8 (2.3, 3.4)	2.7 (2.5,3.5)	0.661
Activated CD8^+^ T cells	6.3 (4.0,9.3)	6.9 (5.3,8.8)	0.719

**Abbreviation:** IQR, Inter-Quartile Range.

**Table 3:** Correlation between the changes in the proportions of lymphocyte subsets in peripheral blood and efficacy after ICI treatment.

Variable	N	Response		Non-response		P value
		Population %		Population %		
No. of cases	61	48	78.69	13	21.31%	
CD3^+^ T cells						0.517
Decrease	28	21	75.00	7	25.00	
Increased	33	27	81.82	6	18.18	
CD3^+^CD4^+^ T cells						0.460
Decrease	32	24	75.00	8	25.00	
Increased	29	24	82.76	5	17.24	
CD3^+^CD8^+^ T cells						0.065
Decrease	24	16	66.67	8	33.33	
Increased	37	32	86.49	5	13.51	
CD4/CD8 ratio						0.732
Decrease	35	27	77.14	8	22.86	
Increased	26	21	80.77	5	19.23	
NK cells						0.202
Decrease	28	20	71.43	8	28.57	
Increased	33	28	84.85	5	15.15	
B cells						0.661
Decrease	49	38	77.55	11	22.45	
Increased	12	10	83.33	2	16.67	
NKT cells						0.561
Decrease	38	29	76.32	9	23.68	
Increased	23	19	82.61	4	17.39	
Ts cells						0.608
Decrease	32	26	81.25	6	18.75	
Increased	29	22	75.86	7	24.14	
Memory T cells						0.016
Decrease	39	27	69.23	12	30.77	
Increased	22	21	77.42	1	22.58	
Activated T cells						0.460
Decrease	32	24	75.00	8	25.00	
Increased	29	24	82.76	5	17.24	
Activated CD8^+^ T cells						0.984
Decrease	28	22	78.57	6	21.43	
Increased	33	26	78.79	7	21.21	

**Table 4:** Correlation between the changes in peripheral blood lymphocyte subset percentages and clinicopathological features after ICI treatment.

	CD3^+^ T cells		CD3^+^CD4^+^ T cells		CD3^+^CD8^+^ T cells		CD4/CD8 ratio		NK cells		B cells		NKT cells		Ts cells		Memory T cells		Activated T cells		Activated CD8^+^ cells	
	**z (after-before)**	**P**	**z (after-before)**	**P**	**z (after-before)**	**P**	**z (after-before)**	**P**	**z (after-before)**	**P**	**z (after-before)**	**P**	**z (after-before)**	**P**	**z (after-before)**	**P**	**z (after-before)**	**P**	**z (after-before)**	**P**	**z (after-before)**	**P**
**Age**		0.123		0.809		0.963		0.646		0.575		0.396		0.303		0.400		0.720		0.049		0.046
≤60 years	1.3		-2.1		1.4		-0.2		3.1		-3.6		-0.4		-2.1		-2.6		0.3		0.7	
>60 years	3.3		-0.7		1.8		-0.3		2.5		-2.4		-0.3		0.2		-2.8		-0.2		-1.2	
**Type of hepatitis**		0.024		0.609		0.933		0.893		0.353		0.101		0.801		0.700		0.147		0.597		0.034
no hepatitis	9.6		-2.4		4.0		-0.3		1.1		-2.2		-0.5		-1.8		3.8		-0.2		-1.1	
hepatitis B	-0.6		1.5		0.9		-0.3		2.9		-3.6		-0.3		0.4		-2.9		0.1		0.7	
**Line of therapy**		0.381		0.879		0.520		0.885		0.568		0.002		0.557		0.729		0.029		0.380		0.469
first line	2.0		-1.2		0.9		-0.3		3.5		-3.9		-0.4		-1.9		-3.5		-0.1		0.4	
later lines	2.3		-2.7		2.6		-0.2		-1.3		-1.7		-0.3		0.2		3.8		0.4		-1.0	
**Distant metastasis**		0.179		0.021		0.647		0.009		0.547		0.018		0.142		0.383		0.994		0.738		0.477
No	4.6		2.5		1.5		0.1		3.0		-4.0		-0.3		0.5		-2.7		-0.2		0.3	
Yes	-2.4		-3.8		1.6		-0.6		-1.3		-2.2		-0.4		-2.0		-2.8		-0.1		0.2	
