# Supplementary material for: The alterations in peripheral lymphocyte subsets predict the efficacy and prognosis of immune checkpoint inhibitors in hepatocellular carcinoma
Source: J Cancer. 2023 Sep 11;14(15):2946–55. doi: 10.7150/jca.88101 (PMC10539567; doi:10.7150/jca.88101)
Supplement: Supplementary file 1 — Supplementary information. [file jcav14p2946s1.pdf]

Supplement: Flow cytometry

The flow experiment steps are as follows:

(1) three flow cytometry tubes were required for each peripheral blood sample and labeled Tube1, Tube2, and Tube3.

(2) Different premixed monoclonal antibodies were added to three tubes, and 50μL of anticoagulated blood was added to each tube.

(3) Mix well and incubate at room temperature in the dark for 15 min.

(4) Add 500μL of hemolysin and mix well with vortex shaking and place in the dark for 15 min.

(5) 1 ml of sheath fluid was added to terminate hemolysis.

(6) Centrifuge the blood sample at 1500 rpm for 5 min and discard the supernatant.

(7) resuspended by adding 500μL of sheath fluid and analyzed by Beckman Coulter Cytometer FC500 flow cytometry.

Antibodies used for flow cytometry were obtained from BD Biosciences (San Jose, CA, USA) and includedCD3-FITC (#555332),CD4-FITC (#550628), CD8-FITC (#555366), CD19-FITC (#555412), CD56-PE (#55664), CD45RO-APC (#559865), CD45RA-PE (#555489), CD38-PE (#555460), FITC/PE/APC isotype controls (#555748; #555749; #555576). Cell Quest software (BD, Franklin Lakes, NJ, USA) was used to analyze the proportion of peripheral blood lymphocyte subsets.
